# Improving carotenoid production in recombinant yeast, *Saccharomyces cerevisiae*, using ultrasound‐irradiated two‐phase extractive fermentation

**DOI:** 10.1002/elsc.202100051

**Published:** 2021-10-29

**Authors:** Ryosuke Yamada, Yorichika Ando, Ryosuke Mitsui, Asuka Mizobata, Shizue Yoshihara, Hayato Tokumoto, Takuya Matsumoto, Hiroyasu Ogino

**Affiliations:** ^1^ Department of Chemical Engineering Osaka Prefecture University Sakai Osaka Japan; ^2^ Department of Biological Science Osaka Prefecture University Sakai Osaka Japan

**Keywords:** carotenoid, extractive fermentation, transcriptome analysis, ultrasound, yeast

## Abstract

Carotenoids are hydrophobic compounds that exhibit excellent bioactivity and can be produced by recombinant *S. cerevisiae*. Irradiating microorganisms with ultrasonic waves increase the productivity of various useful chemicals. Ultrasonic waves are also used to extract useful chemicals that accumulate in microbial cells. In this study, we aimed to improve the carotenoid production efficiency of a recombinant *S. cerevisiae* using an ultrasonic‐irradiation based two‐phase extractive fermentation process. When isopropyl myristate was used as the extraction solvent, a total of 264 mg/L of carotenoid was produced when batches were subjected to ultrasonic‐irradiation at 10 W, which was a 1.3‐fold increase when compared to the control. Transcriptome analysis suggested that one of the reasons for this improvement was an increase in the number of living cells. In fact, after 96 h of fermentation, the number of living cells increased by 1.4‐fold upon irradiation with ultrasonic waves. Consequently, we succeeded in improving the carotenoid production in a recombinant *S. cerevisiae* strain using a ultrasonic‐irradiated two‐phase extractive fermentation and isopropyl myristate as the solvent. This fermentation strategy has the potential to be widely applied during the production of hydrophobic chemicals in recombinant yeast, and future research is expected to further develop this process.

## 1 INTRODUCTION

Given the depletion of the global petroleum resources and the increased focus on their environmental impact, it is now critical to find ways to produce various useful chemicals from renewable resources [1]. *Saccharomyces cerevisiae* has been widely recognized as a safe tool for bioproduction of various chemicals because it has been widely used in the production of various foods and beverages for many years. Given this it is unsurprising that the application of *S. cerevisiae* in the renewable production of useful chemicals has gained in popularity [2].

Carotenoids are hydrophobic compounds that exhibit various colors including yellow, orange, and red, and are used as colorants in foods, improving the body color of farmed fish, and coloring chicken eggs [3]. In addition, carotenoids exhibit excellent bioactivity, such as antioxidant activity; thus, their demand as dietary supplements and cosmetic additives is increasing. The global carotenoid market is expected to reach US$ 1.52 billion by 2021 [4] and is expected to expand in the future.

β‐Carotene is a carotenoid pigment. In addition, it is a precursor of vitamin A and has a high antioxidant effect, and is even a documented anticancer candidate [3]. Carotenoids are currently produced by fermentation using algae and fungi, but they are difficult to cultivate at high density, which contributes to an increase in production costs [3, 4, 5]. Therefore, several efforts have been made to produce these compounds in recombinant *S. cerevisiae*, which is easy to cultivate in industrial settings [6, 7]. In a previous study, we constructed an *S. cerevisiae* strain, YPH499/Mo3Crt79, which expresses the three exogeneous genes, phytoene synthase, lycopene cyclase, and phytoene desaturase, from the naturally carotenoid‐producing yeast *Xanthophyllomyces dendrorhous* at optimal expression levels. This strain produces β‐carotene efficiently [8].

It has been reported that irradiating microorganisms with ultrasonic waves at an appropriate intensity can increase the productivity of various useful chemicals [9, 10]. However, the effect of ultrasonic irradiation on the growth rate and chemical production efficiency of recombinant *S. cerevisiae* has not been widely evaluated. On the other hand, high‐intensity ultrasonic waves are used to extract useful chemicals such as carotenoids, which are produced and accumulated in microbial cells [11, 12]. Therefore, it is likely that the production of carotenoids may be increased by the addition of ultrasonic irradiation at an appropriate intensity during microbial cultivation.

In this study, we aimed to improve carotenoid production in recombinant *S. cerevisiae* using ultrasonic‐irradiated two‐phase extractive fermentation. First, carotenoid production was examined by culturing the β‐carotene‐producing recombinant *S. cerevisiae* in the presence of various organic solvents that act as solvents for the hydrophobic carotenoids and irradiating ultrasonic waves at various intensities. In addition, transcriptome analysis was conducted to evaluate the effects of ultrasonic wave irradiation.

**PRACTICAL APPLICATION**

In this study, hydrophobic carotenoids production in recombinant yeast was successfully improved by using a ultrasonic‐irradiated two‐phase extractive fermentation strategy. When isopropyl myristate was used as the extraction solvent, a total of 264 mg/L of carotenoid was produced when batches were subjected to ultrasonic‐irradiation at 10 W, which was a 1.3‐fold increase when compared to the control. Transcriptome analysis suggested that one of the reasons for this improvement was an increase in the number of living cells. Given the recent developments in synthetic biology, the productivity of hydrophobic high value‐added compounds (e.g., carotenoids and isoprenoids) by recombinant microorganisms continues to improve, and the importance of two‐phase extractive fermentation is increasing. Developed fermentation strategy has the potential to be widely applied during the production of hydrophobic chemicals in recombinant yeast, and future research is expected to develop this process further.

## 2 MATERIALS AND METHODS

### 2.1 Strains, media, and cultivation

The recombinant β‐carotene‐producing yeast strain *S. cerevisiae* YPH499/Mo3Crt79 was used in all our experiments [8]. Briefly, the strain was constructed using the cocktail δ‐integration strategy [13], which allows for the optimization of multiple gene expression. Thus, the strain expresses the three genes, phytoene synthase (*CrtE*), lycopene cyclase (*CrtYB*), and phytoene desaturase (*CrtI*) derived from naturally carotenoid‐producing yeast *X. dendrorhous* at optimal expression levels.

Synthetic dextrose (SD) medium (6.7 g/L yeast nitrogen base without amino acids [Formedium, Norfolk, UK] and 20 g/L glucose) supplemented with appropriate amino acids and nucleic acids was used for pre‐cultivation. Yeast/peptone/dextrose (YPD) medium (10 g/L yeast extract [Formedium], 20 g/L peptone [Formedium], 20 g/L glucose, and 100 mg/L ampicillin sodium salt) with 15 mL of each of the three kinds of organic solvents (dodecane, butyl oleate, or isopropyl myristate) [14] was used in the ultrasonic‐irradiated two‐phase extractive fermentations. Organic solvents were added to dissolve the hydrophobic carotenoids, including β‐carotene, extracted from the cells.

**FIGURE 1:**
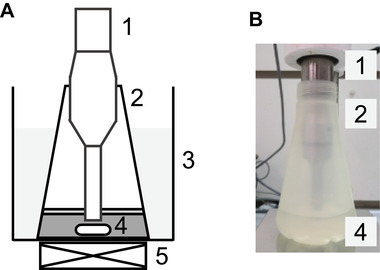
(A) Schematic illustration of the ultrasonic irradiating apparatus. (B) Photo of the ultrasonic irradiating apparatus. 1, ultrasonic homogenizer; 2, flask; 3, water bath; 4, stirrer bar; 5, magnetic stirrer. The water bath and magnetic stirrer are excluded from the photo. In the photo, water is used instead of the medium and the organic solvent

Pre‐cultivation was performed in 5  mL of SD medium using a test tube and a rotary shaker set to 30°C and 150 rpm for 72 h. Ultrasonic‐irradiated two‐phase extractive fermentation was performed in 150 mL YPD medium with 15 mL of organic solvent in a 500 mL flask with baffles and a rotary shaker set to 30°C and 150 rpm. Fermentation was initiated by inoculation (initial OD_600_ = 0.05) of a preculture. During fermentation, intermittent ultrasonic irradiation was performed every 24 h (at 24, 48, and 72 h of fermentation) using an ultrasonic irradiating apparatus (Figure 1). Pre‐determined intensity (10, 80, or 150 W) and 20 kHz ultrasonic irradiation was applied to the culture for 10 min (irradiation 1 s and pause 9 s cycle) using an ultrasonic homogenizer (Sonifier 250DA, Emerson Electric, CT, USA). During ultrasonic irradiation, the culture was maintained at 30°C and stirred with a magnetic stirrer at approximately 150 rpm.

### 2.2 Analysis of carotenoid content and cell viability

The intracellular carotenoid content was determined as described below. A total of 1 mL of culture broth was centrifuged at 10,000 × *g* at room temperature for 5 min and the intracellular carotenoid content in these precipitated cells were isolated using a bead beater homogenizer (μT‐12, Taitec, Saitama, Japan) and hexane as the solvent. Final concentration was determined by colorimetry [8]. β‐Carotene dissolved in hexane was used as the standard.

The extracellular carotenoids dissolved in the organic layer of the fermentation broth were evaluated as described below. A total of 500 μL of the organic layer of the culture broth was centrifuged at 10,000 × *g* and room temperature for 5 min and the concentration of the carotenoids in the supernatant was determined using colorimetry [8]. β‐Carotene was dissolved in the corresponding organic solvent and used as the standard.

The number of living cells was determined using the alkaline methylene blue staining method [15]. Briefly, cells were stained with methylene blue under alkaline conditions, and the number of stained and unstained cells was counted using a hemocytometer (Watson, Tokyo, Japan) and a phase contrast microscope (BX‐3500TPHL; Wraymer, Osaka, Japan).

### 2.3 RNA preparation, library construction, and sequencing

Total RNA was extracted from cells using a NucleoSpin RNA kit (Takara Bio, Otsu, Japan) and then used to prepare a complementary DNA library for next‐generation sequencing using the TruSeq Stranded mRNA Library prep (Illumina, CA, USA) kit and TruSeq RNA Single indexes SetA (Illumina). RNA sequencing was performed using the MiSeq platform (Illumina) and a MiSeq reagent kit v3 (Illumina). The genome sequence of *S. cerevisiae* strain S288c was used as a reference for read mapping via bioinformatics software Geneious (Tomy Digital Biology, Tokyo, Japan). Up‐ and down‐regulated genes were defined as genes satisfying the following conditions: *p* value < 0.05 and absolute value of fold change >1.5.

## 3 RESULTS

### 3.1 Carotenoid production using ultrasonic‐irradiated two‐phase extractive fermentation

**FIGURE 2:**
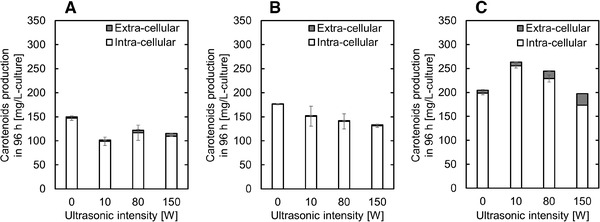
Effect of extraction solvent and ultrasonic intensity on carotenoid production. (A) Dodecane, (B) butyl oleate, and (C) isopropyl myristate were used as extraction solvents. Data represent the mean ± standard deviation (SD) from three independent experiments

First, we investigated the effect of intermittent ultrasonic irradiation on carotenoid production in recombinant yeast. Carotenoid production after 96 h of fermentation was evaluated following intermittent ultrasonic irradiation at various intensities using various extraction solvents (Figure 2).

When dodecane was used as the extraction solvent, approximately 147 mg/L of carotenoids were produced in the cells without ultrasonic irradiation (Figure 2A). In addition, the amount of carotenoid detected in the extracellular fraction was extremely low. No clear relationship was found between ultrasonic intensity and carotenoid production when the ultrasonic waves were applied intermittently. Regardless of the intensity of the ultrasonic waves, the amount of carotenoid produced was smaller than that in the absence of irradiation.

When butyl oleate was used as the extraction solvent, approximately 176 mg/L carotenoid was produced in cells without ultrasonic irradiation (Figure 2B). In addition, the amount of carotenoid detected in the extracellular fraction remained extremely small. The results also suggest that when these fermentations were treated with ultrasonic waves, the total carotenoid production decreased as ultrasonic intensity increased. In addition, as in the case of dodecane, the amount of carotenoid produced was smaller than the control regardless of the intensity of the ultrasonic waves.

When isopropyl myristate was used as the extraction solvent, approximately 199 mg/L of carotenoid was produced in the cells without ultrasonic irradiation (Figure 2C), with up to 6 mg/L of these carotenoids found in the extracellular fraction. Similarly to butyl oleate, our results show that when these fermentations were treated with intermittent ultrasonic waves, the total carotenoid production decreased as ultrasonic intensity increased. However, the total carotenoid production increased when the ultrasonic waves were intermittently irradiated at 10 and 80 W when compared to the non‐irradiated control. When these fermentations were treated with intermittent ultrasonic waves at 10 W, approximately 256 mg/L of carotenoid was produced in the intracellular fraction and approximately 8 mg/L of carotenoid was produced in the extracellular fraction, increasing total carotenoid production to 264 mg/L under these conditions. This represents a 1.3‐fold increase compared to the untreated control.

### 3.2 Effect of intermittent sonication on gene expression

**FIGURE 3:**
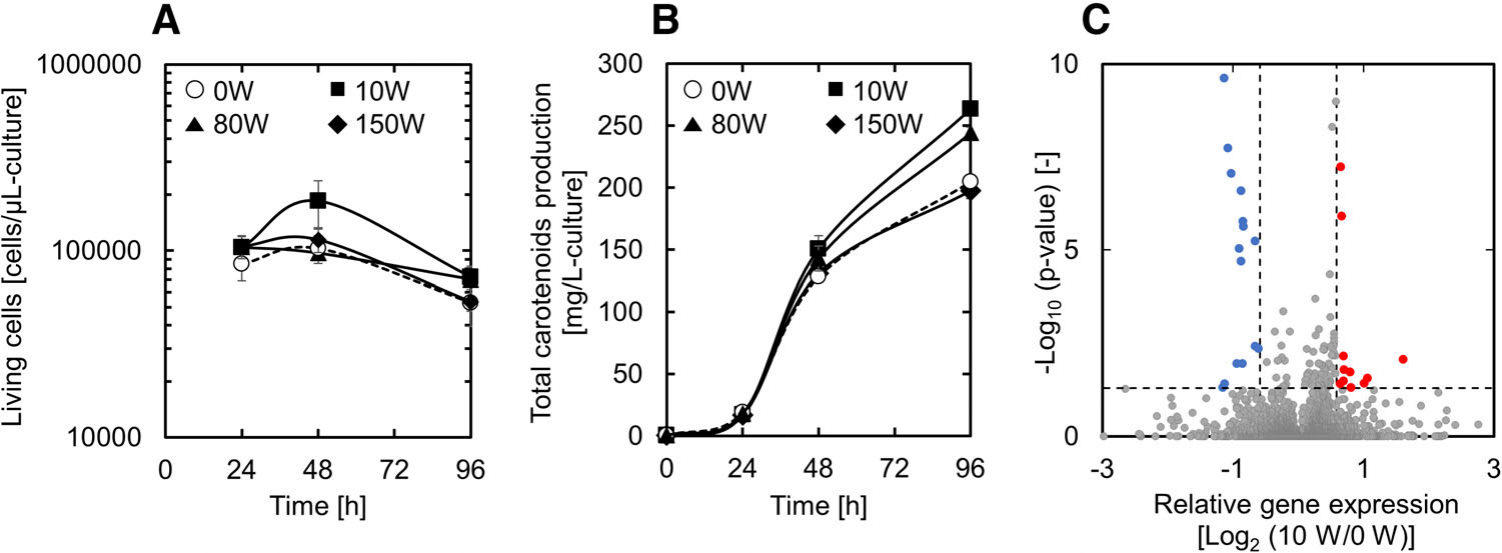
(A) Time course of number of living cells during ultrasonic‐irradiated extractive fermentation with isopropyl myristate. Data represent the mean ± SD from three independent experiments. (B) Time course of number of total carotenoids production during ultrasonic‐irradiated extractive fermentation with isopropyl myristate. Data represent the mean ± SD from three independent experiments. (C) Volcano plot showing changes in gene expression between irradiated and non‐irradiated fermentations. The vertical axis indicates ‐log_10_ (*p*‐value), and the horizontal axis indicates log_2_ fold change. Vertical lines represent fold changes of ±1.5 (log_2_ fold change is approximately ±0.58). The horizontal line represents a *p*‐value of 0.05 (‐log_10_ *p*‐value is approximately 1.30). Red and blue dots represent up‐ and down‐regulated genes, respectively. Gray plot points represent genes whose expression level did not change

As described above, carotenoid production was improved by adding isopropyl myristate and intermittent ultrasonic irradiation at 10 W. Then, we evaluated the time course of the number of living cells and the carotenoids production at the condition (Figure 3A and B). After 48 h of fermentation, the number of viable cells was shown to be 1.9 × 10^5^ cells/μL under 10 W sonication condition, which was higher than other conditions, including untreated condition. After 96 h of fermentation, both 10 and 80 W sonication conditions showed more viable cells than 150 W sonication and untreated conditions. Both after 48 h and after 96 h of fermentation, the most carotenoids were produced under 10 W sonication conditions, followed by 80 W irradiation conditions. On the other hand, no clear change was observed in the state of the culture medium or the morphology of the cells due to the irradiation of ultrasonic waves (Figure 1S and 2S).

To investigate the cause of increased carotenoid production under 10 W sonication condition, we performed a transcriptome analysis of samples from the no treatment control and the 10 W irradiation group using RNA sequencing, and the results are represented by volcano plot (Figure 3C).

Intermediated irradiation at 10 W had a significant effect on 26 genes in these cells, with 11 genes being upregulated and 15 genes being downregulated (Table 1). Genes with improved expression were gene of unknown function (3.05‐fold), *ATX1* (2.08‐fold), which is a cytosolic copper metallochaperone, *SNR41* (2.01‐fold), which is a small nucleolar RNA.

**TABLE 1:** Up‐ and down‐regulated genes following intermittent ultrasonic irradiation

Common name^a^	Systematic name ^a^	Brief description^a^	Fold change
Up‐regulated			
ND	YFR035C	Putative protein of unknown function	3.05
*ATX1*	YNL259C	Cytosolic copper metallochaperone	2.08
*SNR41*	snR41	C/D box small nucleolar RNA	2.01
*RPA12*	YJR063W	RNA polymerase I subunit A12.2	1.75
*SOM1*	YEL059C‐A	Subunit of the mitochondrial inner membrane peptidase	1.73
*AQY1*	YPR192W	Spore‐specific water channel	1.62
*CWP1*	YKL096W	Cell wall mannoprotein	1.61
*ALB1*	YJL122W	Shuttling pre‐60S factor	1.61
*CIT2*	YCR005C	Citrate synthase	1.58
*ICL1*	YER065C	Isocitrate lyase	1.57
*LPX1*	YOR084W	Peroxisomal matrix‐localized lipase	1.56
Down‐regulated			
*ATG29*	YPL166W	Autophagy‐specific protein	0.45
*TAR1*	YLR154W‐C	Protein potentially involved in regulation of respiratory metabolism	0.45
ND	YHR050W‐A	Protein of unknown function	0.46
*RDN18‐2*	RDN18‐2	18S ribosomal RNA	0.47
*RDN18‐1*	RDN18‐1	18S ribosomal RNA	0.49
*MIN6*	YBL039W‐B	Mitochondrial protein of unknown function	0.52
*RDN37‐2*	RDN37‐2	35S ribosomal RNA	0.53
ND	YLR154C‐G	Putative protein of unknown function	0.54
*RDN37‐1*	RDN37‐1	35S ribosomal RNA	0.54
ND	YKR075C	Protein of unknown function	0.55
*RDN25‐2*	RDN25‐2	25S ribosomal RNA	0.55
*RDN25‐1*	RDN25‐1	25S ribosomal RNA	0.56
*COX26*	YDR119W‐A	Subunit of cytochrome C oxidase complex	0.63
*COA2*	YPL189C‐A	Cytochrome oxidase assembly factor	0.63
*MEO1*	YBR126W‐A	Putative protein of unknown function	0.66

ND, not determined.

^a^ According to https://www.yeastgenome.org/ [16].

While the genes with decreased expression included *ATG29* (0.45‐fold), which is an autophagy‐specific protein, *TAR1* (0.45‐fold), which has been linked to the regulation of the respiratory metabolism and gene of unknown function (0.46‐fold).

Some genes with increased gene expression (*ATX1, AQY1*, and *CWP1*) have been reported to be associated with improved cell viability [17, 18, 19]. The result is consistent with the fact that the 10 W sonication condition showed more viable cells than the untreated condition

## 4 DISCUSSION

In this study, we demonstrated that carotenoid productivity in recombinant carotenoid‐producing yeast can be improved by the application of an ultrasonic‐irradiated two‐phase extractive fermentation. We examined three extraction solvents and found that the use of isopropyl myristate resulted in the greatest improvement in carotenoid production. In addition, RNA sequencing revealed that this process upregulated the genes associated with improved cell viability.

In this study, we examined three extraction solvents, dodecane, butyl oleate, and isopropyl myristate, and confirmed that isopropyl myristate was the most suitable for carotenoid production in recombinant yeast (Figure 2). Brennan et al. investigated the organic solvent used in the two‐phase extractive fermentation of monoterpenes in recombinant *S. cerevisiae* [14], and showed that both butyl oleate and isopropyl myristate were promising in terms of yeast biocompatibility, cost of solvent, and water phase separation efficiency. In addition, dodecane is the most widely used solvent in two‐phase extractive fermentation using *Escherichia coli* [20, 21] and *S. cerevisiae* [22, 23]. In this study, one of the root causes of the improved carotenoid production when using isopropyl myristate is likely the high biocompatibility of this solvent compared to other solvents [14]. Another cause is that by using isopropyl myristate as an extraction solvent, it is possible that the produced carotenoids could be efficiently extracted from the cells into the solvent phase. Hydrophobic carotenoids accumulate in cell membranes and/or lipid droplets, and excessive accumulation exhibits cytotoxicity by damaging those membranes and denaturing membrane proteins [24, 25, 26]. In previous studies, isopropyl myristate was used as the extraction solvent in the microbial production of the unstable carotenoid derivative retinoids [20]. It has been reported that retinoids can be efficiently extracted from the cells into the solvent phase by two‐phase fermentation using isopropyl myristate, and it can be produced more efficiently than when dodecane is used as solvent. However, little is known about simultaneous ultrasonic irradiation and two‐phase extractive fermentation. Because ultrasonic waves increase the fluidity of cell membranes [27] and promote emulsification [28], the effects of organic solvents on cells and fermentation could be radically different in systems using ultrasonic waves. By ultrasound‐irradiated two‐phase extractive fermentation, it is speculated that the efficiency of extracting the target substance from the cells by the organic solvent will be increased and the cytotoxicity of the organic solvent will be enhanced. Given the recent developments in synthetic biology, the productivity of hydrophobic high value‐added compounds (e.g., carotenoids and isoprenoids) by recombinant microorganisms continues to improve [29, 30], and the importance of two‐phase extractive fermentation is increasing. Therefore, it is expected that research on suitable organic solvents for two‐phase extractive fermentation and ultrasonic irradiation fermentation will continue to be prioritized in the future.

In this study, carotenoid production by recombinant yeast was improved by adding irradiation with low intensity (10 W) ultrasonic waves, especially when used in combination with isopropyl myristate (Figure 2C). On the other hand, increasing the intensity of ultrasonic waves reduced carotenoid productivity. One of the reasons for the improvement in carotenoid productivity seems to be an increase in the number of viable cells in the fermentation (Figure 3A). Previous studies have reported that irradiating yeast with ultrasonic waves of appropriate intensity reduces the lag phase [31], increases the cell growth rate [32], and final cell mass [33]. Therefore, ultrasonic irradiation fermentation is considered to be widely useful for improving targeted chemical production, especially in the production of growth‐linked chemicals in recombinant yeast. On the other hand, it takes time and effort to determine an appropriate ultrasonic irradiation protocol, and there are still concerns regarding its broad applicability at very large scale. There are many parameters that must be optimized for proper ultrasonic irradiation including, irradiation frequency, total irradiation time, irradiation intensity, and ultrasonic frequency, with each likely to need optimization for each target compound. In addition, various ultrasonic irradiation apparatuses such as horn, bath, and chamber types have been proposed [34]. In previous studies, various parameters and apparatuses were used for yeast cultivation, but there is, at present, no unified approach for this type of production. In the future, it is hoped that more information on ultrasonic irradiation fermentation using yeast will allow for a more unified approach and improved ease of optimization.

Gene expression was not significantly increased or decreased by ultrasound irradiation and relatively few genes made the threshold for differential expression (Figure 3C). Among the genes whose expression levels were increased by ultrasonic irradiation only a handful, including *ATX1*, *AQY1* and *CWP*, had any known function (Table 1). It has been pointed out that all these gene may lead to improved cell viability under stress conditions [17, 18, 19]. Therefore, an increase in the expression levels of these genes may lead to an increase in the number of living cells in these fermentations. On the other hand, *ATG29* and *TAR1* were downregulated under these conditions (Table 1), but the effect of this decrease on cell viability and carotenoid productivity remains unclear.

There are very few reports in which changes in gene expression due to ultrasonic irradiation in yeast have been verified by transcriptome analysis [35, 36]. In addition, the use of two‐phase extractive fermentation in recombinant yeast is still relatively rare. Schults et al. reported the results of their transcriptome analysis of *S. cerevisiae* after 5 days of cultivation with intermittent ultrasonic irradiation [36]. According to this report, only a limited number of genes experienced any type of differential expression, which was consistent with the results of this study. On the other hand, it also reports the results of a metabolome analysis [36] which suggests that although the change in gene expression level was small, ultrasonic irradiation had a significant effect on the major parts of the carbon metabolism including pyrimidine, proline, alanine, aspartate, glutamate, and arginine metabolism. Therefore, it is possible that carotenoid productivity was improved not only by increasing the number of living cells but also by changes at the metabolic level. In future research, it is expected that a more detailed analysis including metabolome and proteome analysis will help to identify the underlying cause of the improved carotenoid productivity associated with ultrasonic‐irradiated two‐phase extractive fermentation and will help to establish a more efficient carotenoid production system using recombinant yeast.

Generally, carotenoids produced by microorganisms accumulate in cells and are extracted into an organic solvent by heating, supercritical carbon dioxide, ultrasonic waves, or the like after fermentation [4, 37, 38]. In addition, excessive accumulation of carotenoids in cells inhibits cell proliferation and carotenoid production [24, 25, 26]. Therefore, in ultrasonic‐irradiated two‐phase extractive fermentation, if the carotenoids can be extracted into an organic solvent with high efficiency without negative effects on cell growth during fermentation, it should improve carotenoid productivity by reducing the inhibitory effects of these compounds and reducing the carotenoid extraction cost from the cells. In previous studies, two‐phase extractive fermentation using microalgae *Dunaliella salina* [39] and cyanobacteria *Synechocystis* sp. [40] was shown to improve carotenoid production. Over 25 wt% and 70 wt% of carotenoids were extracted from *D. salina* and *Synechocystis* sp., respectively, when using two‐phase extractive fermentation and simple agitation. However, in this study, although ultrasonic irradiation was applied intermittently, the percentage of carotenoids produced in the extracellular fraction never exceeded 8.2 wt% (Figure 2C). In *D. salina*, researchers assumed that the improvement in extracellular carotenoid content was directly linked to the improvement of cell permeability due to their contact with the organic solvent and the increase in shear stress associated with increased agitation [39]. However, *S. cerevisiae* are more robust than *D. salina*, and the effects of the organic solvent and shear stress on the cells is much smaller, this might explain why the carotenoid extraction efficiency remained low despite their exposure to ultrasonic waves. In the filamentous fungus *Monascus*, which is known to have a robust cell structure, similar to *S. cerevisiae*, it has been reported that the addition of a surfactant improves cell permeability and improves pigment extraction in two‐phase extractive fermentation [41]. This method may also be applied to the ultrasonic‐irradiated two‐phase extractive fermentation proposed in this study. In the future, it is expected that the optimum ultrasonic irradiation conditions (ultrasonic irradiation conditions and apparatus) and the use of chemicals to improve cell permeability, such as surfactants, will be examined to improve both the extracellular extraction efficiency and productivity of carotenoids.

## 5 CONCLUDING REMARKS

In this study, we improved carotenoid production in a recombinant yeast strain using ultrasonic‐irradiated two‐phase extractive fermentation and isopropyl myristate as the solvent. Transcriptome analysis suggests that one of the reasons for this improvement is an increase in the number of living cells. Although promising, there are a few issues that need to be resolved before this can be widely applied. These include the optimization of the ultrasonic irradiation conditions and evaluating the scalability of these approaches. This preliminary data supports the use of ultrasonic‐irradiated two‐phase extractive fermentation in the production of hydrophobic chemicals by recombinant yeast, and future research is expected to develop this technology and bring it to wide scale application.

## CONFLICT OF INTEREST

The authors have declared no conflict of interest.

## Supporting information

Supporting information

## Data Availability

The data that support the findings of this study are available from the corresponding author upon reasonable request.
