# Supplementary material for: Improving carotenoid production in recombinant yeast, Saccharomyces cerevisiae, using ultrasound‐irradiated two‐phase extractive fermentation
Source: Eng Life Sci. 2021 Oct 29;22(1):4–12. doi: 10.1002/elsc.202100051 (PMC8727735; doi:10.1002/elsc.202100051)
Supplement: Supplementary file 1 — Supporting information [file ELSC-22-4-s001.pdf]

Improving carotenoid production in recombinant yeast, *Saccharomyces cerevisiae*, using ultrasound-irradiated two-phase extractive fermentation

Supplementary Figure

Ultrasonic intensity [W]	Cultivation time [h]		
	24	48	96
0	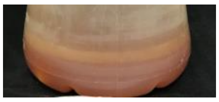	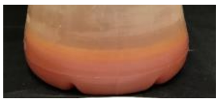	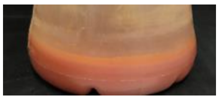
10	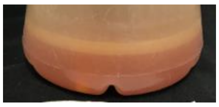	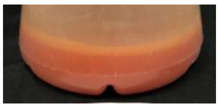	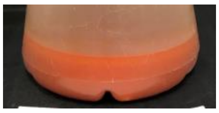
80	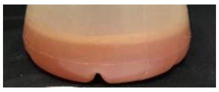	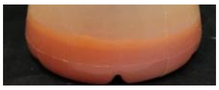	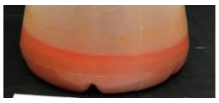
150	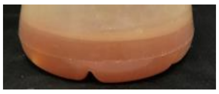	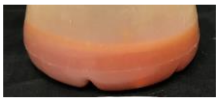	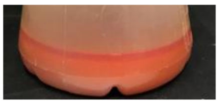

Fig. 1S. State of the culture medium during ultrasonic-irradiated extractive fermentation with isopropyl myristate.

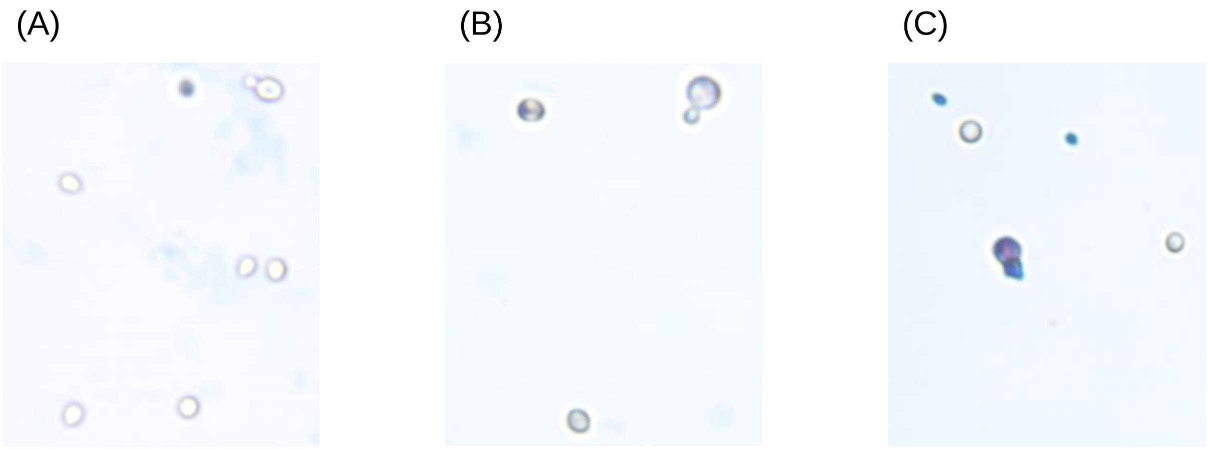

Fig. 2S. Morphological change of the cells during ultrasonic-irradiated extractive fermentation with isopropyl myristate. A) After 24 h fermentation. B) After 48 h fermentation. C) After 96 h fermentation. The images were acquired by a 400x phase contrast microscope after staining with methylene blue.
